# Small Pore-Forming Toxins Different Membrane Area Binding and Ca^2+^ Permeability of Pores Determine Cellular Resistance of Monocytic Cells †

**DOI:** 10.3390/toxins13020126

**Published:** 2021-02-09

**Authors:** Yu Larpin, Hervé Besançon, Victoriia S. Babiychuk, Eduard B. Babiychuk, René Köffel

**Affiliations:** Institute of Anatomy, University of Bern, 3012 Bern, Switzerland; yu.larpin@ana.unibe.ch (Y.L.); herve.besancon@ana.unibe.ch (H.B.); viktoria.babiychuk@ana.unibe.ch (V.S.B.); edik.babiychuk@ana.unibe.ch (E.B.B.)

**Keywords:** pore-forming toxin, pore channel diameter, plasma membrane repair, Ca^2+^ oscillations

## Abstract

Pore-forming toxins (PFTs) form multimeric trans-membrane pores in cell membranes that differ in pore channel diameter (PCD). Cellular resistance to large PFTs (>20 nm PCD) was shown to rely on Ca^2+^ influx activated membrane repair mechanisms. Small PFTs (<2 nm PCD) were shown to exhibit a high cytotoxic activity, but host cell response and membrane repair mechanisms are less well studied. We used monocytic immune cell lines to investigate the cellular resistance and host membrane repair mechanisms to small PFTs lysenin (*Eisenia fetida*) and aerolysin (*Aeromonas hydrophila*). Lysenin, but not aerolysin, is shown to induce Ca^2+^ influx from the extracellular space and to activate Ca^2+^ dependent membrane repair mechanisms. Moreover, lysenin binds to U937 cells with higher efficiency as compared to THP-1 cells, which is in line with a high sensitivity of U937 cells to lysenin. In contrast, aerolysin equally binds to U937 or THP-1 cells, but in different plasma membrane areas. Increased aerolysin induced cell death of U937 cells, as compared to THP-1 cells, is suggested to be a consequence of cap-like aerolysin binding. We conclude that host cell resistance to small PFTs attack comprises binding efficiency, pore localization, and capability to induce Ca^2+^ dependent membrane repair mechanisms.

## 1. Introduction

Plasma membranes are composed of a diverse set of lipids and provide a functional barrier that separates the inside of a cell from the outside environment [1]. Pathogens produce pore-forming toxins (PFTs) that disrupt the plasma membrane and induce lysis or apoptosis of host cells. PFTs are secreted in monomers from bacteria to the extracellular milieu where they will bind to receptors on host cell membranes. Receptors for PFTs are diverse and include lipid species, sugars or cell surface proteins, i.e., cholesterol, and/or GPI-anchored proteins [2]. For example, pneumolysin, secreted by *Streptococcus pneumoniae*, binds specifically to the cell membrane’s cholesterol, whereas lysenin (from *Eisenia fetida)* preferentially binds to areas with ordered sphingomyelin within the host cell membrane [3,4]. In contrast, aerolysin from *Aeromonas hydrophila* binds to GPI-anchored proteins expressed on host cells [5]. PFTs monomers, after binding to their receptors, assemble into multimers and form a functional trans-membrane pore. PFTs perforation of the plasma membrane can lead to a massive influx of components from the extracellular space, i.e., Ca^2+^- or K^+^-ions, and a loss of cytoplasmatic content [1]. Cells injured by PFTs are not necessarily sentenced to death, but possess membrane repair mechanisms to reseal the damaged plasma membrane region and ensure survival [6,7].

PFTs are a diverse toxin family that differ in size of the formed pore channel and structural compositions (i.e., α-helices or β-barrels for their trans-membrane elements) [1]. Interestingly, different pore-forming toxins can induce a variety of cellular responses, independent of their common feature of building a trans-membrane pore [8]. Differences in PFTs consequences are suggested to be attributed to the size of the pore, disturbances of cellular ion homeostasis, and differential efficiency of plasma membrane repair mechanisms in host cell types [9]. For example, the inability to rapidly reseal the damaged plasma membrane leads to detrimental high intracellular Ca^2+^ levels due to influx through toxin pores, which induces apoptosis [7,10].

Recently, we showed that pneumolysin, a cholesterol dependent cytolysin of approximately 26 nm pore channel diameter (PCD), displays differences in toxicity depending on the immune cell type [6,11]. A rapid rise of intracellular Ca^2+^, triggered by pneumolysin pores, recruits Ca^2+^ sensitive annexin family proteins to the plasma membrane to plug the pores [7,12]. Subsequently, damaged plasma membrane areas are released to the extracellular environment in the form of microvesicles, a process termed “shedding” [13]. Shedding after PFTs attack was shown to be operational in various immune cell types, although with varying efficiency. Monocytic cells were able to shed a significant higher number of repair-microvesicles as compared to lymphoid cells, which displayed poor shedding capability [6]. In line with the importance of the shedding process for membrane repair, monocytic cells are more resistant to lysis and cell death induced by pneumolysin, when compared to lymphoid cells.

Small PFTs are produced by many pathogens, i.e., aerolysin from *Aeromonas hydrophila* or lysenin from earthworm *Eisenia fetida.* It was suggested that small pores exhibit their relatively high toxicity due to less efficient membrane repair mechanisms [1]. However, membrane repair efficacy of small PFTs remains to be investigated. Aerolysin and lysenin belong to the family of β-barrel PFTs. They differ in their host cell receptors, but both toxins form pore channels of approximatively 2 nm size in diameter [14,15,16]. Aerolysin requires GPI-anchored proteins in the host cell membrane to form functional pores that are enriched in lipid microdomains (= lipid rafts) in the plasma membrane [17]. Lysenin induced toxicity depends on ordered sphingomyelin in the plasma membrane, also found predominantly in lipid rafts [18]. Recently, it was shown that the toxin pore-selectivity for extracellular Ca^2+^ differ between lysenin (Ca^2+^ -permeable pores) and aerolysin (Ca^2+^ -inefficient pores) [9]. Interestingly, aerolysin activity was shown to induce the release of Ca^2+^ from intracellular stores in host cells [19].

In the present study, we investigated cellular survival mechanisms and toxicity of small toxin pores, i.e., aerolysin and lysenin, for monocytic immune cells. We find for both toxins that THP-1 cells are more resistant as compared to U937 cells. Moreover, we show that lysenin pores, although smaller in size as compared to pneumolysin, can induce the Ca^2+^ dependent membrane repair mechanism. In contrast, aerolysin attack does not induce the Ca^2+^ dependent membrane repair by annexin protein family members in these cells, suggesting that a rise of Ca^2+^ concentrations by release from intracellular stores is not sufficient to activate such cell repair mechanisms.

## 2. Results

### 2.1. Aerolysin and Lysenin Differentially Bind to Monocytic Cell Lines

**Figure 1:**
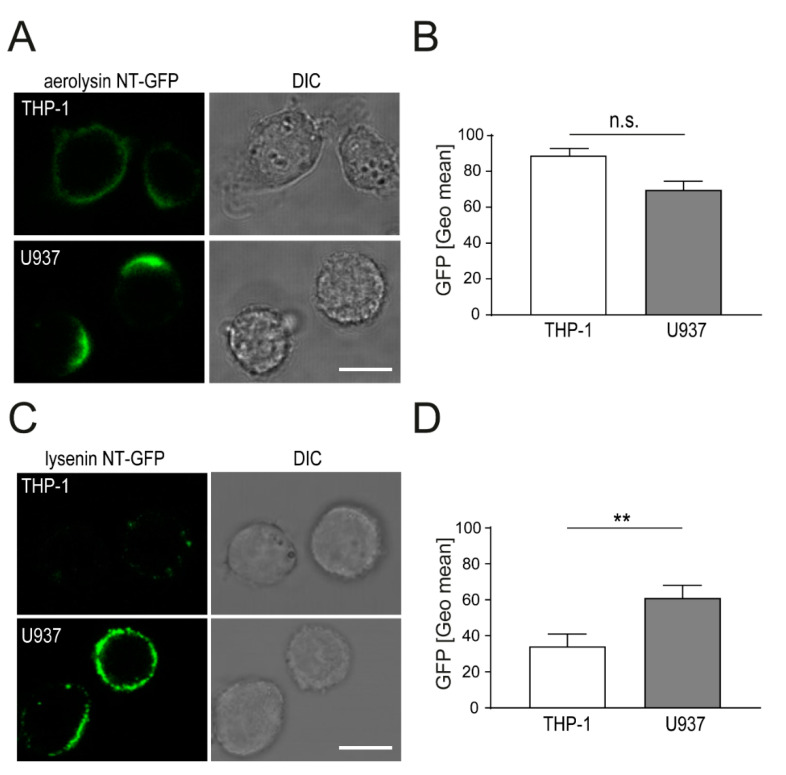
Differential binding of aerolysin NT-GFP and lysenin NT-GFP to monocytic cells. (**A**) THP-1 and U937 cells were incubated with aerolysin NT-GFP for 15 min. Binding of aerolysin NT-GFP (green) was assessed by confocal microscopy (bar 10 µm); (**B**) cells were incubated as in (**A**), washed, and immediately analyzed by flow cytometry (FACS). Mean of GFP intensities, corrected for background fluorescence, ± SEM is shown (n = 3); (**C**) THP-1 and U937 cells were incubated with lysenin NT-GFP for 15 min. Binding of lysenin NT-GFP (green) was assessed by confocal microscopy (bar 10 µm); (**D**) cells were incubated as in (**C**), washed, and immediately analyzed by FACS. Mean of GFP intensities, corrected for background fluorescence, ± SEM is shown (n = 3, ** *p* < 0.005, n.s. = Not Significant).

Plasma membrane binding by PFTs monomers is the initial step, which induces the formation of stable trans-membrane pores. Presumably, detrimental PFTs action depends on efficiency of toxin binding (limiting factor: Toxin receptor abundance on host cell membranes) and pore assembly. We determined the binding efficiency of aerolysin (receptor: GPI-anchored proteins) and lysenin (receptor: Ordered sphingomyelin) binding to monocytic cell lines. U937 and THP-1 cells were incubated with non-toxic green fluorescent protein (NT-GFP) fusion protein, aerolysin NT-GFP for 15 min and analyzed by confocal microscopy [16]. Binding of aerolysin NT-GFP occurred more rapidly in U937 cells as compared to THP-1 cells. Interestingly, aerolysin NT-GFP showed a polarized binding pattern and formed a cap in U937 cells (Figure 1A). In contrast, THP-1 cells showed a uniform aerolysin NT-GFP binding pattern of the whole plasma membrane. PFTs were suggested to cluster membrane areas, thus influencing the distribution of lipids within the plasma membranes [20]. To assess if GPI-anchored proteins are distributed in U937 cells in a polarized pattern, we performed live binding assay with aerolysin NT-GFP. Immediate cap-like binding of aerolysin NT-GFP is observed in U937 cells within the first minute (Video S1A). No cap-like signal of aerolysin NT-GFP is observed in THP-1 cells, immediately after stimulation or at later time points (T = 15 min) (Video S1B). Quantification of amounts of cell bound aerolysin NT-GFP by flow cytometry showed no significant differences in U937 compared to THP-1 cells (Figure 1B).

Lysenin NT-GFP is a non-toxic form of full-length lysenin and is frequently used as a fluorescent probe for ordered sphingomyelin platforms on the plasma membrane [21]. We used this fluorescent probe to analyze the binding efficiency of lysenin to THP-1 and U937 cells. Lysenin NT-GFP shows enhanced binding to U937 cells as compared to THP-1 cells after 15 min (Figure 1C). Lysenin NT-GFP signal is distributed in a spotty pattern on the plasma membrane of U937 and THP-1 cells. Interestingly, no polarized capping of lysenin NT-GFP occurs in U937 cells as compared to aerolysin NT-GFP (Figure 1A). Significantly higher amounts of lysenin NT-GFP are bound to U937 cells as compared to THP-1 cells (Figure 1D). Interestingly, both NT-GFP fusion proteins were suggested to detect lipid rafts in the plasma membrane, yet they display a distinct localization pattern in U937 cells (polarized capping for aerolysin NT-GFP versus spotty uniform distribution for lysenin NT-GFP).

### 2.2. Localization of Aerolysin Differs in U937 Versus THP-1 Cells and Causes Rapid Permeabilization of U937 Cells

**Figure 2:**
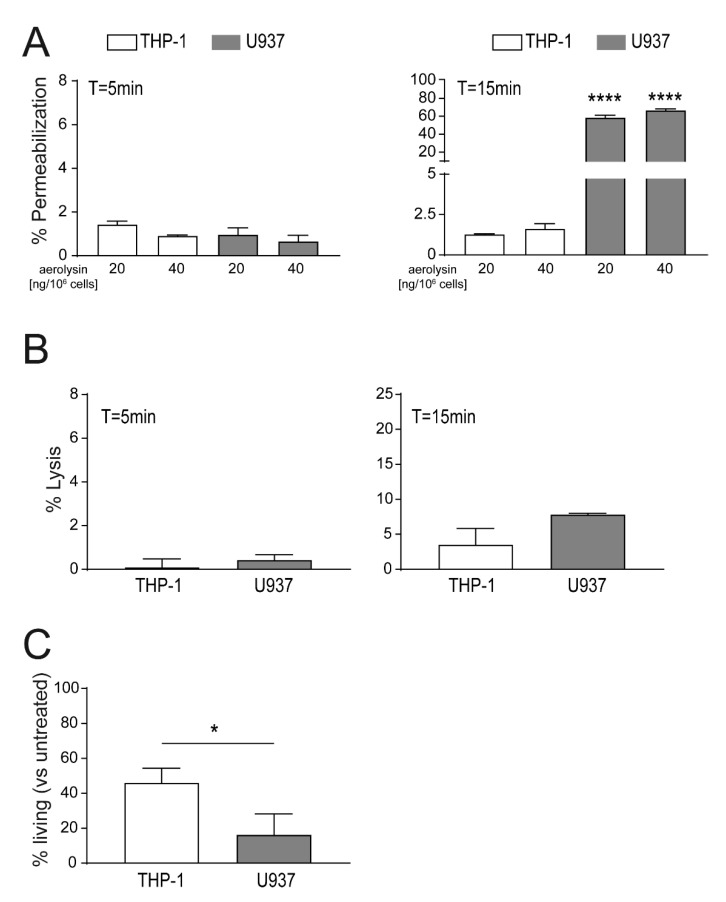
Enhanced permeabilization to aerolysin treatment of U937 versus THP-1 cells. (**A**) Equal numbers of U937 and THP-1. cells were stimulated with increasing concentrations of aerolysin [ng/10^6^ cells] and cell permeabilization was monitored using propidium iodide influx after 5 min (left panel) or 15 min (right panel) by FACS; (**B**) cells were stimulated with 20 ng aerolysin/10^6^ cells and cell lysis after 5 min (left panel) and 15 min (right panel) were analyzed by FACS; (**C**) cells were stimulated with 2 ng aerolysin/10^6^ cells for 30 min. Subsequently, the unbound toxin was removed, and the cells were recovered in full RPMI media for 24 h. Percentages of living cells at T = 24 h were assessed using the Alamar blue^®^ assay. Mean ± SEM is shown (n = 3, * *p* < 0.05, **** *p* < 0.0001).

Active toxin pores in the plasma membranes can induce influx of extracellular milieu into the cell, which can lead to a rise of intracellular ion concentrations, i.e., Ca^2+^. Changes in intracellular Ca^2+^ levels were shown to trigger a repair machinery to re-seal the damaged plasma membrane areas. However, cells exposed to high PFTs concentrations are not able to repair their plasma membrane and will ultimately be lysed. For cellular permeabilization assays, we titrated aerolysin to sublytic concentrations, to avoid immediate lysis of target cells. Concentrations of aerolysin between 20 ng to 80 ng/10^6^ cells were shown to induce below 10% lysis at the endpoint of the assay (T = 15 min, Figure S1A). Permeabilization studies of THP-1 and U937 cells in response to 20 ng/10^6^ cells aerolysin showed below 2% of permeabilized cells after 5 min for THP-1 cells or U937 cells (Figure 2A). Increment of aerolysin concentrations by 2-fold (40 ng/10^6^ cells) did not significantly increase immediate permeabilization of U937 or THP-1 cells after 5 min (Figure 2A). Prolonged exposure to aerolysin (T = 15 min) enhanced the permeabilization of U937 cells only (60% permeabilization), whereas permeabilization of THP-1 stayed low (<2% permeabilized cells) (Figure 2A). Interestingly, permeabilization in response to higher aerolysin concentrations (40–80 ng/10^6^ cells) is not significantly different in U937 nor THP-1 cells (Figure S1A). Aerolysin induced lysis of THP-1 and U937 cells shows moderate levels at 5 min (<1% lysed cells) and 15 min (<10% lysed cells) post-toxin stimulation (Figure 2B). Interestingly, increment of aerolysin concentrations (up to 80 ng/10^6^ cells) did not significantly increase cell lysis in both cell types (THP-1 < 10% lysed cells, U937 < 15% lysed cells; Figure S1A). These data suggest that the levels of GPI-anchored proteins expressed on the surface of U937 or THP-1 cells limit pore formation and thus detrimental effects of aerolysin. THP-1 cells display a higher resistance to aerolysin as compared to U937 which may be attributed to membrane repair efficiency. Moreover, these data suggest that an increased sensitivity in U937 cells could be attributed to the polarized localization of aerolysin pores at a specific area of the plasma membrane.

Immediate toxin permeabilization and lysis assays showed differences in resistance to aerolysin in U937 versus THP-1 cells. Cellular survival assays analyze membrane repair capacity and thus long-term survival of cells. In contrast to immediate toxin assays where we tested effects of up to 80 ng aerolysin/10^6^ cells, sublytic concentrations of aerolysin are tested. Stimulation of U937 and THP-1 cells with 4 ng aerolysin/10^6^ cells induced < 90% of cell death after 24 h (Figure S1B). Lower concentrations of aerolysin (2 ng/10^6^ cells) showed significantly reduced survival for U937 cells (<20% living cells as compared to the untreated controls). In contrast, THP-1 cells showed a higher survival rate (>40% living cells) after 24 h (Figure 2C). Very low aerolysin concentrations, 1 ng or 0.5 ng/10^6^ cells, allowed respective survival of 75% and 95% of stimulated U937 cells (Figure S1B). In contrast, THP-1 cells were not affected by these low aerolysin concentrations (100% surviving cells) (Figure S1B). Together, these data showed that U937 cells are more susceptible to aerolysin as compared to THP-1 cells, in terms of the immediate toxin effects (i.e., permeabilization) and long-term survival assays.

### 2.3. Enhanced Binding of Lysenin to U937 Causes Increased Cell Death as Compared THP-1 Cells

**Figure 3:**
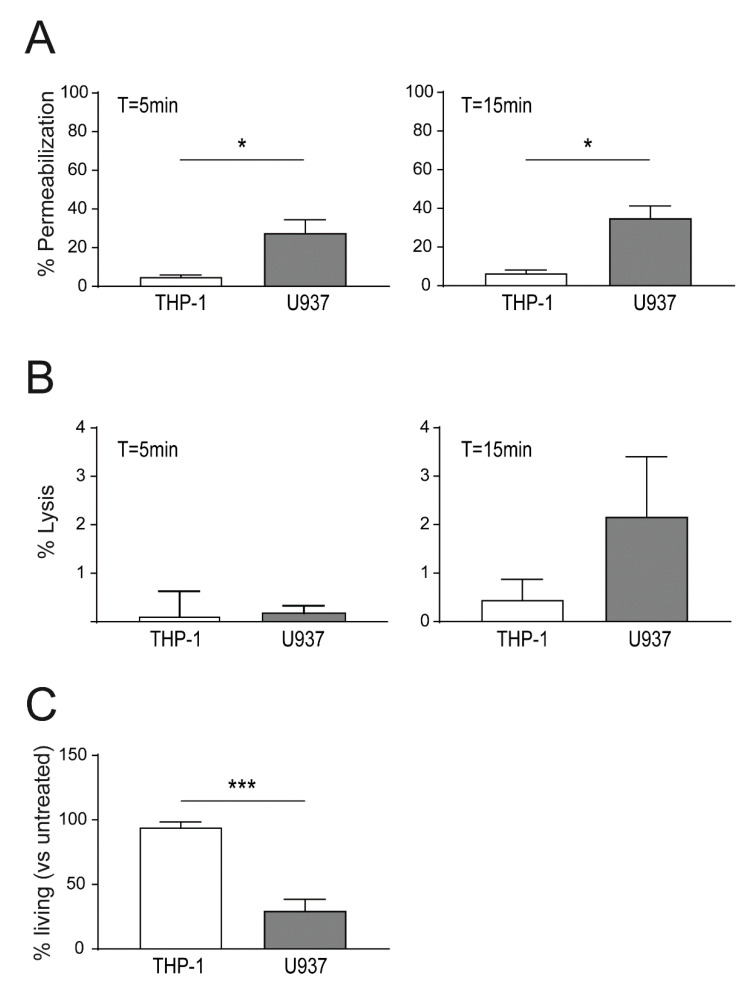
Increased sensitivity to lysenin of U937 versus THP-1 cells. (**A**) Equal numbers of U937 and THP-1 cells were stimulated with 13.75 ng lysenin/10^6^ cells and cell permeabilization was monitored using propidium iodide influx after 5 min (left panel) or 15 min (right panel) post toxin addition by FACS; (**B**) cells were stimulated with 13.75 ng lysenin/10^6^ cells and cell lysis after 5 min (left panel) and 15 min (right panel) were analyzed by FACS; (**C**) cells were stimulated 32 ng lysenin/10^6^ cells for 30 min. Subsequently, the unbound toxin was removed and the cells were recovered in full RPMI media for 24 h. Percentages of living cells at T = 24 h were assessed using the Alamar blue^®^ assay. Mean ± SEM is shown (n = 3, * *p* < 0.05, *** *p* < 0.001).

Sublytic lysenin concentrations (13.75 ng/10^6^ cells; < 1% lysed cells after 5 min), were used to determine permeabilization in U937 versus THP-1 cells. After 5 min of toxin stimulation, THP-1 cells showed a significantly lower permeabilization as compared to U937 cells (< 5% versus 27% of permeabilized cells; Figure 3A). At T = 15 min both cell lines showed a steady 20–25% increase of permeabilized cells. Percentages of lysed cells after stimulation with 13.75 ng lysenin/10^6^ cells remained at low levels (<2%) at 5 min and 15 min post-stimulation (Figure 3B). 

In survival assays, cells were challenged with lysenin for 15 min, and after removal of unbound toxin, cells were allowed to recover for 24 h. After challenge with 32 ng lysenin/10^6^ cells, low cell death in THP-1 cells was detected (94.3% surviving cells at T = 24 h; Figure 3C). Survival rates of THP-1 cells in response to lysenin started to decline with 128 ng lysenin/10^6^ cells (approx. 80% surviving cells) and dropped below 35% surviving cells with 256 ng lysenin/10^6^ cells (Figure S1C). In contrast, U937 cells showed a higher toxin susceptibility with only 30% surviving cells at 32 ng lysenin/10^6^ cells (Figure 3C). A 2-fold increase of lysenin to 64 ng/10^6^ cells reduced the U937 survival rate to 4%, and higher concentrations (128 ng or 256 ng lysenin/10^6^ cells) resulted in 100% dead cells after 24 h (Figure S1C). At equal lysenin concentrations, THP-1 cells viability was unaffected (~90%; 64 ng and 128 ng) or showed survival rates of ~50% (256 ng lysenin/10^6^ cells) (Figure S1C). Taken together, these data show a high susceptibility of U937 cells to lysenin attack as compared to THP-1 cells.

### 2.4. Plasma Membrane Perforation and Subsequent Intracellular Ca^2+^ Level Changes Differ in Lysenin Versus Aerolysin Attack

**Figure 4:**
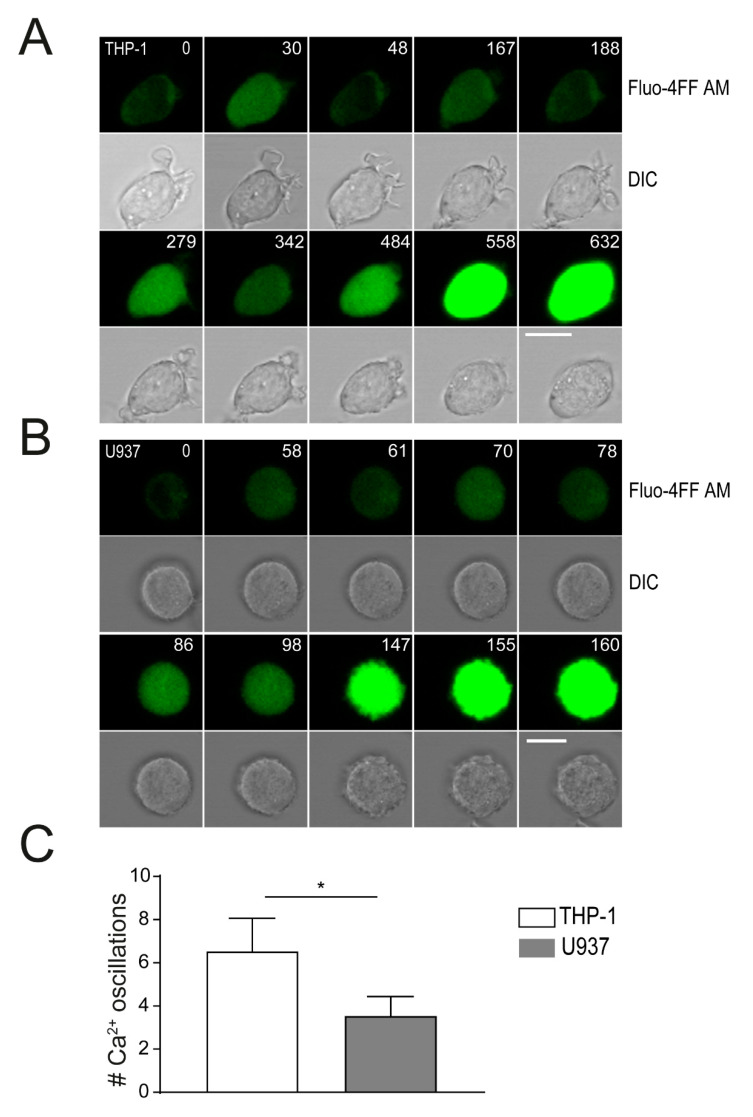
Intracellular Ca^2+^ increase in THP-1 versus U937 cells after stimulation with lysenin. Equal numbers of U937 and THP-1 were loaded with 5 μM Fluo-4FF AM and simulated with 1 µg/ml lysenin (T = 0 s). Changes in intracellular Ca^2+^ concentrations in (**A**) THP-1; (**B**) U937 cells were monitored using serial imaging by confocal microscopy (T = s; bar 10 µm). Images of one representative experiment are shown (n = 3); (**C**) quantification of rounds of Ca^2+^ oscillations was performed by confocal microscopy, counting 10 cells each. Mean ± SEM from 3 independent experiments is shown (* *p* < 0.05).

Permeabilization of the plasma membrane leads to intracellular Ca^2+^ increase, which is followed by the removal excess of Ca^2+^ from the cytoplasm to intracellular stores or through ion pumps to the extracellular space [10]. Ca^2+^ influx through toxin pores can induce a monophasic rise of intracellular Ca^2+^, that activates a cellular repair machinery consisting of proteins of the annexin family. To monitor changes in intracellular Ca^2+^ after PFTs stimulation in monocytic immune cells, we loaded cells with Fluo-4FF AM, stimulated with toxins and analyzed by confocal microscopy. Stimulation of THP-1 and U937 cells with lysenin (1 µg/ml) rapidly induced an equal oscillatory increase of Fluo-4FF AM signal at T = 30–60 s (Figure 4A,B). Oscillations of Fluo-4FF AM signal intensities were shown to occur either after active transport of Ca^2+^ to the extracellular space by calcium channels or by Ca^2+^ release from intracellular storage compartments, i.e., the endoplasmatic reticulum [10]. A stop in Ca^2+^ oscillations and final high fluorescent intensities (saturated) of Fluo-4FF AM occurred after 2.5 min in U937 cells or 9 min in THP-1 cells. Quantification of Ca^2+^ oscillation events until the endpoint (saturated Fluo-4FF AM fluorescence) showed a significantly lower number in U937 cells (~3.5 oscillations) as compared to THP-1 cells (~6.5 oscillations) (Figure 4C, Video S2A,B). These data confirm a high susceptibility of U937 cells to lysenin attack.

For aerolysin stimulation (400 ng/ml), minor changes in Fluo4-FF AM signals could be detected in THP-1 cells (33–56 s) and in U937 cells (75–85 s). However, a single major Fluo-4FF AM signal pulse was shown in U937 (T = 7 min) and in THP-1 cells (T = 3.5 min) (Figure S2A,B). These data suggest an inefficient Ca^2+^ permeability of aerolysin pores. A high fluorescent Fluo-4FF AM signal at later time points may results from a loss of integrity of the cell membrane (and thus Ca^2+^ influx), and not by influx through aerolysin pores.

In Ca^2+^ free conditions, THP-1 cells stimulated with lysenin displayed intracellular Ca^2+^ oscillations within the first 100 s and were lysed after this time point. In contrast, Ca^2+^ oscillations were not observed in U937 cells in the absence of extracellular Ca^2+^ (Figure S3A,B). Cells in Ca^2+^-free conditions stimulated with aerolysin showed no intracellular Ca^2+^ oscillations, but a small increase of intracellular Fluo4-FF AM signal in U937 (T = 14 min) and THP-1 cells (T = 13 min), accompanied by major cell damage in both cell lines (Figure S4A,B).

Perforation of the plasma membrane by large PFTs can induce a loss of cytoplasmatic content [22]. Loss of cytoplasmic content after stimulation with small PFTs was analyzed with GFP expressing THP-1 or U937 cells. Interestingly, lysenin and aerolysin pores do not allow leakage of cytoplasmic GFP in THP-1 (Figure S5A,B) and U937 cells (Figure S6A,B).

Annexin family members are Ca^2+^-sensitive membrane repair proteins that respond to a rise of cytoplasmatic Ca^2+^ concentrations by translocations to the plasma membrane followed by resealing of the damaged membrane parts by relocation to the cytoplasm [8]. We used an annexinA2-GFP construct (that was shown to be the most sensitive annexin to Ca^2+^ changes) [8], to visualize these membrane repair processes in monocytic immune cells in response to PFTs, i.e., aerolysin and lysenin. Stimulation of annexinA2-GFP transfected cells with lysenin induced translocations of annexinA2-GFP from the cytoplasm to the inner layer of the plasma membrane and back to the cytoplasm. Several cycles of these translocations occurred in THP-1 cells (Video S3A). In contrast, aerolysin stimulation showed no back and forth cycles of cytoplasm to plasma membrane translocation (Video S3B). Lysenin-stimulated U937 cells also showed a rapid initiation of annexinA2-GFP translocation to the plasma membrane and back to the cytosol (Video S4A). In analogy to THP-1 cells, no activation of annexinA2-GFP is induced in U937 cells in response to aerolysin (Video S4B). No repetitive annexinA2-GFP plasma membrane translocations in U937 or THP-1 cells occurred with decreasing aerolysin concentrations as low as 100 ng/10^6^ cells (data not shown). These data suggest that no sufficient Ca^2+^ influx for annexin-repair machinery activation after aerolysin attack occurs. Moreover, minor Ca^2+^ influx induced by aerolysin does not activate the annexin-mediated membrane repair machinery in monocytic immune cell lines U937 and THP-1.

## 3. Discussion

Pneumolysin, a cholesterol-dependent large PFT (PCD ~26 nm) from *Streptococcus pneumoniae*, was shown to induce different responses in different immune cell types [6,11]. Although plasma membrane cholesterol levels did not differ significantly between cell types, the lipid distribution in the plasma membrane affected PFTs activity and thus sensitivity to pneumolysin attack. In addition, efficacy of Ca^2+^ initiated membrane repair mechanisms were shown to positively correlate with cellular resistance to toxin damage. 

Here, we investigated the toxicity of aerolysin from *Aeromonas hydrophila* and lysenin from *Eisenia fetida*, which are two small PFTs of approximatively 2 nm PCD [14,23]. We find that monocytic immune cells U937 and THP-1 display different susceptibilities against these toxins. U937 cells were rapidly permeabilized by lysenin and aerolysin and cell viability after stimulation with low concentrations of lysenin or aerolysin, was shown to be reduced. In contrast, THP-1 cells were less permeabilized by lysenin or aerolysin and thus showed a higher viability after toxin stimulation as compared to U937 cells.

Aerolysin and lysenin were shown to engage different host cell receptors for binding and pore assembly. Aerolysin binds to GPI-anchored proteins [24] and lysenin binds to ordered sphingomyelin patches in the plasma membrane of target cells [23]. Both, GPI-anchored proteins and ordered sphingomyelin patches were suggested to be enriched in membrane microdomains (= lipid rafts) [17,18]. Lipid rafts play an important role in cellular functions, i.e., signaling [25]. However, lipid rafts were also shown to serve as docking platforms for toxin monomers, and to potentiate toxin pore oligomerization [26,27]. We show a homogenous, spotty binding of lysenin (NT-GFP) to U937 and THP-1 cells, suggesting an equal distribution of membrane lipid rafts in both cell types. However, binding efficiency of lysenin to U937 exceeds THP-1 cells by a factor of 2. Thus, U937 cells may possess a higher number of lipid rafts (or ordered sphingomyelin patches) in their cell membrane as compared to THP-1 cells [28]. In line with the enhanced lysenin binding, U937 cells showed a rapid permeabilization and a low cell viability after lysenin attack. THP-1 cells showed a significant lower permeabilization and a high cell viability after lysenin stimulation. In contrast, aerolysin showed equal binding efficiency to U937 and THP-1 cells. However, U937 cells were shown to be highly sensitive to aerolysin attacks as compared to THP-1 cells. Interestingly, aerolysin (NT-GFP) showed a strong cap-like binding to U937 cells, whereas in THP-1 cells the toxin distribution on the plasma membrane was more homogenous. Aerolysin localization on the plasma membrane may facilitate the process of pore formation in U937 cells. In line with that, microdomains were shown to act as concentration platforms to facilitate aerolysin pore formation [17]. Moreover, pneumolysin, which also targets lipid rafts, was suggested to be capable of triggering the formation of stable microdomains within cellular membranes [29]. Cap-like binding of aerolysin to U937 cells was shown to occur with fast kinetics. In contrast, prolonged exposure of THP-1 cells with aerolysin NT-GFP did not induce cap-like localization. Thus, it is unlikely that aerolysin triggers the formation of stable membrane platforms in host cell membranes that eventually would lead to enhanced detrimental toxin action. However, in U937 cells, the concentration of aerolysin pores in a cap-like area on the plasma membrane may inhibit efficient membrane repair. It will be interesting to further investigate the causalities of membrane pore localization and pore removal/membrane repair in future experiments.

Non-physiological intracellular Ca^2+^ concentrations were shown to be important for initiation of Ca^2+^ dependent membrane repair by annexin protein family members [8]. Large PFTs, i.e., pneumolysin, were shown to be permeable to Ca^2+^ and thus damaged membrane areas were rapidly repaired by Ca^2+^ dependent repair [13]. We show that small PFT lysenin triggered Ca^2+^ influx in THP-1 and U937 cells. In addition, multiphasic intracellular Ca^2+^ level changes were shown in response lysenin attack that may result from cell protective mechanisms: (i) Removal of excess intracellular Ca^2+^ through activated Ca^2+^ channels or (ii) rapid elimination of lysenin pores by Ca^2+^ dependent membrane repair mechanisms [6,10]. Streptolysin O and α-toxin were shown to also induce a release of Ca^2+^ from intracellular stores [19]. After removal of extracellular Ca^2+^, lysenin simulation induced minor fluctuations of Fluo-4FF AM signal in U937 and THP-1 cells, suggesting a release of Ca^2+^ from intracellular stores. In contrast, aerolysin stimulation only triggered minor Ca^2+^ influx, and in Ca^2+^ depleted conditions, minor Ca^2+^ release from intracellular stores was observed only at late time points. Small PFTs damage was shown to be difficult to repair, which may result from insufficient plasma membrane repair initiation/efficiency [1]. The membrane repair protein annexinA2 was shown to translocated to the plasma membrane at low (<1 µM) intracellular Ca^2+^ changes, i.e., rise in Ca^2+^ through PFTs induced membrane perforation [8,30]. Moreover, Ca^2+^ release from intracellular stores was shown to induce a partial translocation of annexinA2 to the plasma membrane [31]. AnnexinA2 translocations to the plasma membrane was shown in U937 and THP-1 cells in response to lysenin attack only. These data show that Ca^2+^ flux through small PFTs, i.e., lysenin, is sufficient to activate annexin-mediated membrane repair in these cells. Lack of translocations of annexinA2-GFP after aerolysin stimulation suggested inefficient Ca^2+^ flux through the toxin pores. The non-activation of Ca^2+^ sensitive membrane repair proteins, i.e., annexinA2, may potentiate aerolysin toxicity towards monocytic immune cell lines. Lysenin or aerolysin induced Ca^2+^ release from intracellular stores was shown to not be sufficient to activate translocations of annexinA2-GFP in U937 or THP-1 cells.

Thus, monocytic immune cell lines showed different sensitivities to small PFTs through different mechanisms: (i) Target receptor abundance, (ii) toxin pore localization in specific plasma membrane areas, and (iii) Ca^2+^ permeability of toxin pores. 

## 4. Materials and Methods

### 4.1. Cells and Reagents

U937 (ATCC CRL-1593.2, Manassas, USA) and THP-1 (ATCC TIB-202, Manassas, USA) were maintained in RPMI1640 medium supplemented with 10% heat-inactivated fetal bovine serum (FBS) (Gibco, Life Technologies, Paisley, UK) and 1% penicillin-streptomycin (Gibco, Life Technologies, Paisley, UK) in 5% CO_2_ at 37 °C. Lentiviral constructs for stable transfection of annexin A2-GFP or GFP-alone control cells were a kind gift of Prof. Katia Monastyrskaya (Urology Research Laboratory, Department of Clinical Research, University of Bern, Bern, Switzerland). Lentiviral infections were performed according to established protocols [32], and transfected cells were sorted for expression of GFP with a FACS AriaII (BD Instruments, San Jose, CA, USA). Wild-type aerolysin, lysenin, and aerolysin NT-GFP were a kind gift of Mircea-Ioan Iacovache (Institute of Anatomy, University of Bern, Bern, Switzerland). Recombinant lysenin NT-GFP was produced and purified in our laboratory as described previously [29]. 

### 4.2. Permeabilization and Lysis Assays

Assays were performed as previously described with minor changes [33]. In brief, 2 × 10^6^ cells in Ca^2+^ Tyrode’s buffer (140 mM NaCl, 5 mM KCl, 1 mM MgCl_2_, 10 mM glucose, 10 mM HEPES (pH 7.4), 2.5 mM Ca^2+^) were incubated with 20 μg/mL propidium iodide (PI) (Sigma-Aldrich, Munich, Germany). After addition of aerolysin or lysenin, cells were transferred to 96-well plates. Numbers of PIpositive cells were recorded with a CytoFLEX (Beckman Coulter, Krefeld, Germany) at 0, 5, and 15 min post-toxin addition. Specific lysis was determined as follows:

(1) $$\%\mathrm{Specific}\;\mathrm{Lysis}\;=\frac{\left(\%\mathrm{PI}\;\mathrm{HighExperimental}\;-\%\mathrm{PI}\;\mathrm{HightControl}\right)}{\left(100-\%\mathrm{PI}\;\mathrm{HightControl}\right)}$$

Permeabilized cells were determined using PI Low instead of PI High populations as described [33].

### 4.3. Viability Assays

For the viability experiments, 2 × 10^6^ cells were washed in Ca^2+^ Tyrode’s buffer and incubated with PFTs as indicated in Ca^2+^ Tyrode’s buffer (final volume 1 ml) for 30 min at room temperature (aerolysin was activated with trypsin prior). In all cell survival experiments, cells were pelleted after stimulation with PFTs and grown in 3 ml RPMI 1640 medium with penicillin/streptomycin and 10% FBS for 24 h. For measuring viability, 0.1 volume of alamar Blue reagent (Thermo Fisher Scientific, Waltham, MA, USA) was pipetted directly into 100 μL aliquots of the cells in culture medium and incubated for 3–4 h at 37 °C under protection from direct light. Fluorescence emission was measured with a microplate reader (SpectraMax Gemini; Molecular Devices, San Jose, CA, USA) at 590 nm with an excitation wavelength of 560 nm. 

### 4.4. Toxin Binding Assays 

Aerolysin NT-GFP was a kind gift of Mircea-Ioan Iacovache (Institute of Anatomy, University of Bern, Bern, Switzerland). Lysenin NT-GFP was a kind gift of Prof. Toshihide Kobayashi (Laboratory of Bioimaging and Pathologies, University of Strasbourg, France) [16]. For microscopic analysis, 1.5 × 10^6^ cells were pretreated with 0.96 µg aerolysin NT-GFP or 3.42 µg lysenin NT-GFP in Ca^2+^-Tyrode’s buffer, washed with Ca^2+^-Tyrode’s buffer, transferred to chamber slides (Ibidi, Gräfelfing, Germany) and allowed to settle for ∼5 min. Sequential images were recorded with an LSM 880 system (Zeiss, Jena, Germany) with a 63× oil-immersion lens. Quantitative analysis of aerolysin NT-GFP or lysenin NT-GFP binding was performed by flow cytometry with a LSRII (BD Biosciences, Franklin Lakes, NJ, USA).

### 4.5. Measurements of Cytosolic Ca^2+^

For cellular Ca^2+^ measurements, 3 × 10^6^ cells were incubated with 5 μM Fluo-4FF AM (Invitrogen, Carlsbad, CA, USA) in 5% CO_2_ at 37 °C for 30 min, washed with Ca^2+^-Tyrode’s buffer, and resuspended in RPMI1640 medium supplemented with 10% FBS and 1% penicillin-streptomycin. Before transfer to chamber slides, cells were washed, and resuspended in Ca^2+^-Tyrode’s buffer. For assays in Ca^2+^ free conditions, cells were incubated in with Na-Tyrode’s buffer without Ca^2+^ supplemented with 5 mM EGTA. Before microscopy, EGTA was removed by a washing step with Na-Tyrode’s buffer without EGTA. Sequential images were recorded with an LSM 880 system (Zeiss, Jena, Germany).

### 4.6. Statistics

Numerical data are expressed as mean values together with the standard error. The statistical analyses were performed using GraphPad Prism7 software (GraphPad Software, La Jolla, CA, USA). Significant differences are marked with asterisks (* *p* < 0.05, ** *p* < 0.005, *** *p* < 0.001, **** *p* < 0.0001).

## Data Availability

Data sharing not applicable.

## Supplementary Materials

The following are available online at https://www.mdpi.com/2072-6651/13/2/126/s1, Figures S1–S6 and Video S1A–S4B.
