# Supplementary material for: Small Pore-Forming Toxins Different Membrane Area Binding and Ca2+ Permeability of Pores Determine Cellular Resistance of Monocytic Cells
Source: Toxins (Basel). 2021 Feb 9;13(2):126. doi: 10.3390/toxins13020126 (PMC7914786; doi:10.3390/toxins13020126)
Supplement: Supplementary file 1 [file toxins-13-00126-s001.zip › toxins-1065707 sup crosscheck.docx]

Supplementary Material: Small Pore-Forming Toxins Different Membrane Area Binding and Ca2+ Permeability of Pores Determine Cellular Resistance of Monocytic Cells

Yu Larpin, Hervé Besançon, Victoriia S. Babiychuk, Eduard B. Babiychuk and René Köffel

**Video S1.** (**A**) U937 or **(B)** THP**-**1 cells in Ca**-**Tyrode**’**s buffer were seeded to chamber slides and stained with 1**.**7μg aerolysin NT GFP/10^6^ cells aerolysin NT GFP at T = 0 min. Movies were recorded using a LSM880 confocal microscope and processed using ZenBlue software (Zeiss, Germany) (bar 10μm; time = min).

**Video S2.** (**A**) U937 or (**B**) THP-1 cells pre-treated with 5μM Fluo-4FF AM priory to stimulation with 222 ng lysenin / 106 cells at T = 0 min. Movies were recorded using a LSM880 confocal microscope and processed using ZenBlue software (Zeiss, Germany) (bar 10μm; time = min).

**Video S3.** THP-1 annexinA2-GFP cells in Ca-Tyrode’s buffer were seeded to chamber slides and stimulated with (**A**) 166 ng lysenin/10^6^ cells or (**B**) 176 ng aerolysin / 106 cells at T = 0 min. Movies were recorded using a LSM880 confocal microscope and processed using ZenBlue software (Zeiss, Germany) (bar 10μm; time = min).

**Video S4.** U937 annexinA2-GFP cells in Ca-Tyrode’s buffer were seeded to chamber slides and stimulated with (**A**) 166 ng lysenin/10^6^ cells or (**B**) 176 ng aerolysin / 106 cells at T = 0 sec. Movies were recorded using a LSM880 confocal microscope and processed using ZenBlue software (Zeiss, Germany) (bar 10μm; time = sec/min).

**Figure S1.** (**A**) Equal numbers of U937 and THP-1 cells were stimulated with increasing concentrations of aerolysin [ng/10^6^ cells] and cell permeabilization, and lysis was monitored using propidium iodide influx after 15 min post-toxin addition by FACS. (**B**–**C**) Cells were stimulated with increasing concentrations of aerolysin (**B**) or lysenin (**C**) for 30 min. Subsequently, the unbound toxin was removed, and the cells were recovered in full RPMI media for 24 h. Percentages of living cells at T = 24 h were assessed using the Alamar blue® assay. Mean ± SEM is shown (*n* = 3).

**Figure S2.** (**A**) THP-1 and (**B**) U937 cells were loaded with 5 μM Fluo-4FF AM and Scheme 400 ng aerolysin/ml (T = 0 sec) in the presence of Ca2+. Changes in intracellular Ca2+ concentrations were monitored using serial imaging by confocal microscopy (T = sec; bar 10μm). Images of one representative experiment are shown (*n* = 3).

**Figure S3.** Equal numbers of (**A**) THP-1 and (**B**) U937 cells were loaded with 5 μM Fluo-4FF AM and simulated with 1 μg lysenin/ml (T = 0 sec) in Ca2+ depleted conditions. Changes in intracellular Ca2+ concentrations were monitored using serial imaging by confocal microscopy (T = sec; bar 10μm). Images of one representative experiment are shown (*n* = 3).

**Figure S4.** (**A**) THP-1 and (**B**) U937 cells were loaded with 5 μM Fluo-4FF AM and simulated with 400 ng aerolysin/ml (T = 0 sec) in Ca2+ depleted conditions. Changes in intracellular Ca2+ concentrations were monitored using serial imaging by confocal microscopy (T = sec; bar 10 μm). Images of one representative experiment are shown (*n* = 3).

**Figure S5.** GFP (green) expressing THP-1 cells were simulated with (**A**) lysenin (166 ng/10^6^ cells; T = 0 min) or (**B**) aerolysin (176 ng/106 cells; T = 0 min) in the presence of 4 μg/ml propidium iodide (red). Cells were monitored using serial imaging by confocal microscopy up to 12 min post-toxin addition (T = min; bar 10μm). Influx of propidium iodide shows permeabilization and cell death. Images of one representative experiment are shown (*n* = 3).

**Figure S6.** GFP (green) expressing U937 cells were simulated with (**A**) lysenin (166 ng / 10^6^ cells; T = 0 min) or (**B**) aerolysin (176 ng/10^6^ cells; T = 0 min) in the presence of 4 μg/ml propidium iodide (red). Cells were monitored using serial imaging by confocal microscopy up to 12 min post-toxin addition (T = min; bar 10μm). Influx of propidium iodide shows permeabilization and cell death. Images of one representative experiment are shown (*n* = 3).
